# Comparative Analysis of Oral and Oropharyngeal Mucosal Lesions of American Tegumentary Leishmaniasis and Other Infectious Granulomatous Diseases and Squamous Cell Carcinoma

**DOI:** 10.3390/pathogens15010101

**Published:** 2026-01-17

**Authors:** Clarissa Souza Mota Reis, João Gustavo Corrêa Reis, Raquel de Vasconcellos Carvalhaes de Oliveira, Cláudia Maria Valete, Fátima Conceição-Silva

**Affiliations:** 1 Evandro Chagas National Institute of Infectious Diseases (INI), Oswaldo Cruz Foundation (Fiocruz), Av. Brasil 4365, Rio de Janeiro 21040-360, RJ, Brazil; 2 Laboratory of Immunoparasitology, Oswaldo Cruz Institute (IOC), Oswaldo Cruz Foundation (Fiocruz), Av. Brasil 4365, Rio de Janeiro 21040-900, RJ, Brazil; 3 Department of Bronchoesophagolaryngology and Head and Neck Surgery, Bonsucesso Federal Hospital, Av. Londres 616, Rio de Janeiro 21041-020, RJ, Brazil; 4 Department of Otorhinolaryngology and Ophthalmology, Faculty of Medicine, Federal University of Rio de Janeiro, Av. Carlos Chagas Filho 373, Edifício do CCS, Bloco K, Rio de Janeiro 21941-971, RJ, Brazil

**Keywords:** oral manifestation, American tegumentary leishmaniasis, paracoccidioidomycosis, sporotrichosis, histoplasmosis, tuberculosis, leprosy, squamous cell carcinoma

## Abstract

American tegumentary leishmaniasis (ATL) and other infectious granulomatous diseases (IGDs) may present with oral/oropharyngeal mucosal lesions (OOPML). IGD-OOPML can result from fungal, parasitic, or bacterial infections, and squamous cell carcinoma (SCC) represents the main differential diagnosis. ATL, other IGD, and SCC share overlapping clinical and epidemiological features, making diagnostic suspicion challenging. This study compared sociodemographic and clinical characteristics among ATL, other IGD, and SCC. Descriptive, comparative, and multivariable logistic regression analyses were performed. Among 7551 patients, 213 met inclusion criteria (83-SCC and 130-IGD). Except for smoking, which differed only between ATL and SCC, most IGD parameters were similar. Male patients predominated in all groups. SCC patients were significantly older (*p* < 0.001) and had a shorter median disease duration (*p* = 0.007). The presence of pain increased the odds of SCC-OOPML by 3.96 times (95% CI 1.97–12.51). SCC patients were more likely to present lesions in a single subsite, either the oral cavity or oropharynx. Painful, ulcerated, or exophytic lesions favored SCC diagnosis, whereas infiltrative, granular, or mulberry-like lesions, involvement of multiple subsites, or associated nasal and laryngeal lesions suggested IGDs. Although clinical differentiation remains difficult, these findings may support early diagnostic suspicion, prompt treatment, and reduced sequelae.

## 1. Introduction

Infectious granulomatous diseases (IGD) can cause lesions of varied aspects in the oral cavity and oropharynx. They are so named owing to the histopathological presence of a chronic inflammatory response characterized by the formation of granulomas [1]. The etiology can be fungal, parasitic, or bacterial; in particular, the diseases of American tegumentary leishmaniasis (ATL), paracoccidioidomycosis (PCM), sporotrichosis (SP), histoplasmosis (HP), tuberculosis (TB), and leprosy (LP) stand out [2,3,4,5,6,7].

Leishmaniasis is an infectious parasitic disease, with a worldwide distribution, caused by the protozoan *Leishmania* spp., which can present clinically as visceral or tegumentary disease. Tegumentary disease can be further classified as either the cutaneous form or mucosal form [8]. In particular, 90% of its mucocutaneous presentations (ATL) are observed in South America [8]. In recent years, a downward trend in the number of ATL cases has been observed in the Americas, but despite this, 34,954 cases were reported to the Pan American Health Organization (PAHO) in this region in 2023, and Brazil was the country with the highest number of cases—4528 new cases were reported in 2024, with 7% corresponding to the mucosal form [9,10]. ATL can be found in all five regions of Brazil, and the northern region is the most affected. *Leishmania* (*Viannia*) *braziliensis* is the main species and is distributed throughout the country; *Leishmania* (*Viannia*) *guyanensis* and *Leishmania* (*Leishmania*) *amazonensis* are predominant in the north region (e.g., Amazon) [11]. The parasite is transmitted through the bite of infected female sandflies. The most common clinical presentation is one or a few painless rounded or oval-shaped ulcers with an erythematous, indurated base with a firm consistency and well-defined, raised borders with a reddish base and coarse granulations on exposed areas of the skin [12]. The mucosal forms of ATL are usually secondary to skin lesions, affecting 3–10% of patients, and manifest as destructive lesions located in the mucous membranes of the upper airways. In some patients, the mucosal lesion occurs by extension from an adjacent (contiguous) cutaneous lesion, or on the exposed semimucosa, such as the lip. It is also possible to have a concomitance of skin and mucosal lesions in the same patient. The onset of symptoms is insidious, with few complaints. The nasal mucosa is the most affected area, and oral and oropharyngeal mucosal lesions (OOPML) are concomitant to nasal lesions in most cases [12,13,14]. The precise mechanisms of development of mucosal lesions are still under investigation, but results have demonstrated early dissemination of the parasites from the skin and the mucosal location, even in the absence of clinical lesions [15,16,17].

PCM is a systemic mycosis caused by fungi of the genus *Paracoccidioides*, which is endemic to Latin America, with 80% of cases registered in Brazil [5,18]. Infection occurs through the inhalation of propagules, which are generally associated with handling soil contaminated with the fungus, such as during agricultural activities and earthmoving [5]. Most cases are observed in the southeastern, southern, and midwestern regions of Brazil, although the number of cases has increased in the northern region due to deforestation [19,20]. These areas are also endemic for ATL, and when mucosal lesions are present [10,11,18], conclusive diagnostics become difficult if the etiological agent is not demonstrated. The lungs are the most frequently affected organs, and OOPML are observed in 50–75% of patients with PCM, mainly in the chronic form of the disease, even if no signs of pulmonary disease are detected [21,22]. SP is a subcutaneous mycosis with global distribution, with areas of high endemicity in Latin America [23,24]. *Sporothrix* spp. are the etiological agents of the disease, and the type of transmission and clinical presentations seem to be related to the species [24,25]. Transmission occurs through inoculation of the fungus into the skin or mucous membranes via trauma resulting from accidents with thorns, straw, or wood splinters; contact with decaying vegetation; or scratches or bites from infected animals, with cats being the most common source of infection. The cutaneous form is the most frequent, and two main clinical presentations are observed: the lymphocutaneous form and the single lesion, known as the fixed form of the mycosis [2,23]. The clinical appearance can be easily confused with ATL, specifically the sporotrichoid cutaneous lesion and the single cutaneous ATL lesion. Although involvement of the oral or oropharyngeal mucosa in SP is rare and occurs mainly in immunocompromised individuals [2,26], diagnostic difficulty is further increased in the presence of mucosal lesions, as these cannot be visually distinguished from ATL. This difficulty is increased because, in many regions, the endemic areas of SP and ATL overlap [23,25,27]. HP is another globally distributed disease caused by fungi of the genus *Histoplasma*, although it is highly endemic to the Ohio and Mississippi river valleys (USA) and in areas of South and Central America [28]. Infection occurs through the inhalation of fungal spores commonly found in soil contaminated with bird and bat droppings. OOPML in HP are rare but are more frequent in immunocompromised patients and in the disseminated form of the disease [29,30]. Although HP is less frequently reported, this mycosis may share endemic areas with ATL, SP, and PCM.

TB and LP are bacterial IGDs that can manifest with OOPML. TB is caused by *Mycobacterium tuberculosis* and affects the lungs in particular. The transmission occurs when active TB patients expel bacteria into the air (e.g., by coughing) [31]. OOPML are usually concomitant with a pulmonary focus and can be seen in 0.1–5% of patients [3,32,33]. The number of new TB cases decreased globally in 2024 after 3 years of increases due to disruptions to diagnosis and treatment during the COVID-19 pandemic [31]. LP is a chronic disease caused by *M. leprae* that affects the skin, peripheral nerves, upper respiratory tract mucosa, and eyes. It is believed that the disease is transmitted through contaminated saliva droplets or nasal secretions after close and prolonged contact with patients with untreated leprosy [34]. South-East Asia accounts for 70% of the cases [35]. OOPML occur more frequently in patients with the lepromatous form and seem to be related to a longer duration of disease evolution and late implementation of treatment [6,36,37]. As discussed for SP, HP, and PCM, TB and LP may also be present in regions endemic for ATL, increasing exposure to different sources of infection and complicating differential diagnoses.

Neoplasms are one of the main differential diagnoses of OOPML [4,38,39]. A total of 496,200 patients were diagnosed with oral cavity, lip, and oropharyngeal cancer worldwide in 2022 [40]. Oral cavity cancer accounts for approximately 50% of all head and neck cancers, and squamous cell carcinoma (SCC) is the most frequent histological type [41,42]. Cancers in the oral cavity are often related to the use of tobacco (both smoked and smokeless), alcohol, and areca nut. Pharyngeal cancers are only partly caused by these factors, but cancers of the oropharynx—such as those at the base of the tongue and in the tonsils—are strongly linked to infection with human papillomavirus (HPV), especially HPV16 and HPV18. Lip cancers are connected to ultraviolet (UV) radiation and tobacco exposure, with the specific cause differing by location: the inner lip is more related to tobacco use, while the outer lip is more related to UV exposure [40].

OOPML of IGD and SCC share epidemiological and clinical aspects, and despite having some specific and distinct histopathological findings, it is noteworthy that histopathological studies are not consistently available across all regions, especially in those far from major referral centers and in remote areas. Lesions of both groups can be ulcerated, infiltrative, or exophytic, and may affect individuals of the same sex and similar age groups, which hinders diagnostic suspicion [3,4,43]. ATL, PCM, SP, HP, TB, and LP are not usually included in the scope of SCC-OOPML differential diagnosis. The endemic regions for these IGDs frequently overlap, and even in these areas, many health professionals are not familiar with these conditions. However, professionals all over the world should be aware of these potential diagnoses, particularly given globalization and traveling [2,18,30,44]. As IGDs and SCC require specific therapeutic regimens, enhancing our knowledge about OOPML could lead to faster diagnosis and implementation of treatment, avoiding complications and sequelae. The aim of the present study was to compare the socio-demographic and clinical aspects of ATL, other IGDs, and SCC OOPML and to present factors that may aid in differential diagnoses.

## 2. Materials and Methods

This study was designed as a retrospective cross-sectional analysis of medical records from patients presenting with OOPML treated at the Otorhinolaryngology Service of the Evandro Chagas National Institute of Infectious Diseases (INI-Fiocruz), Rio de Janeiro, Brazil, between January 2005 and December 2017. The study period and general clinical workflow were similar to those previously described by Reis et al. (2024) [45].

A total of 7551 medical records were reviewed, and patients diagnosed with ATL, other IGDs (PCM, SP, HP, TB, and LP) and SCC with confirmed OOPML were eligible for inclusion. Patients with alternative diagnoses, incomplete diagnostic investigation, inconclusive examinations, or loss to follow-up before diagnostic confirmation were excluded, following criteria adapted from our previous study [45].

Clinical and sociodemographic data, including age, sex, race, educational level, smoking and alcohol use, lesion characteristics, anatomical location, pain complaint, and duration of lesion evolution, were extracted from medical records and entered into a dedicated database for analysis. Data collection procedures were standardized according to institutional clinical protocols previously reported [45].

Diagnostic confirmation was based on a combination of clinical evaluation, patient history, laboratory tests (serology, direct examination, or culture), histopathological examination of biopsy specimens from oral, oropharyngeal, or other affected sites, and/or complete remission of lesions after disease-specific treatment. These diagnostic criteria follow the same principles adopted in our earlier publication, but were applied here to address a distinct comparative research question.

For analytical purposes, this study has included the following definitions:

ATL OOPML: Comprises OOPML of ATL patients.
IGD OOPML: Comprises OOPML of all diagnosed IGD, including ATL cases (ATL, PCM, SP, HP, TB, and LP).
SCC OOPML: Comprises OOPML of SCC patients.

OOPML were classified according to morphological aspects as ulcerated, exophytic, infiltrative, granular, hyperemic, or mulberry-like lesions, using definitions adapted from Reis et al. (2016) [46] and maintained for consistency with prior investigations. Lesion location was categorized as oral cavity only, oropharynx only, or combined oral and oropharyngeal involvement, and further subdivided into specific anatomical subsites based on the TNM classification and International Agency for Research on Cancer guidelines, with minor adaptations as previously described.

The duration of evolution was defined as the interval (in months) between patient-reported lesion onset and diagnostic confirmation. Information on dental status, vitamin D levels, detailed immunological parameters, and non-HIV causes of immunosuppression was not systematically available due to the retrospective nature of the study.

Statistical analyses were performed using SPSS version 16.0 (SPSS Inc., Chicago, IL, USA). Comparative analyses were conducted for the ATL OOPML versus SCC OOPML and IGD OOPML versus SCC OOPML groups. The simple frequencies of categorical variables are described, and summary measures are given in terms of minimum, maximum, mean ± standard deviation (SD), median, and interquartile range (IQR) for continuous variables. The association between categorical variables was verified by Pearson’s chi-square test or Fisher’s exact test (when expected count < 5). The Shapiro–Wilk normality test indicated a deviation from normality for the variable ‘time of evolution’. The Mann–Whitney U test was used to compare the median times of evolution, whereas a t-test was used to compare the mean ages. Owing to theoretical importance, the variables ‘general location’ (oral only, oropharyngeal only, and oral/oropharyngeal), ‘more than one subsite per patient’, and ‘pain complaint’ were selected, and ‘age’, ‘sex’, and ‘time of evolution’ were used to build logistic models according to the outcome “diagnostic status” (1 = SCC OOPML, 0 = IGD OOPML). Additionally, we included an interaction term between ‘general location’ and ‘more than one subsite per patient’ in the multiple logistic model. The crude and adjusted effects (single and multiple covariate models) were examined using odds ratios (OR) and their 95% confidence intervals (CI). The other variables were not included in the analysis due to the small number of cases in each category. *p*-values of <0.05 indicated statistically significant differences.

This study was approved by the Research Ethics Committee of INI-Fiocruz (protocol No. 759873179.0000.5262) and conducted in accordance with the Declaration of Helsinki.

## 3. Results

**Figure 1:**
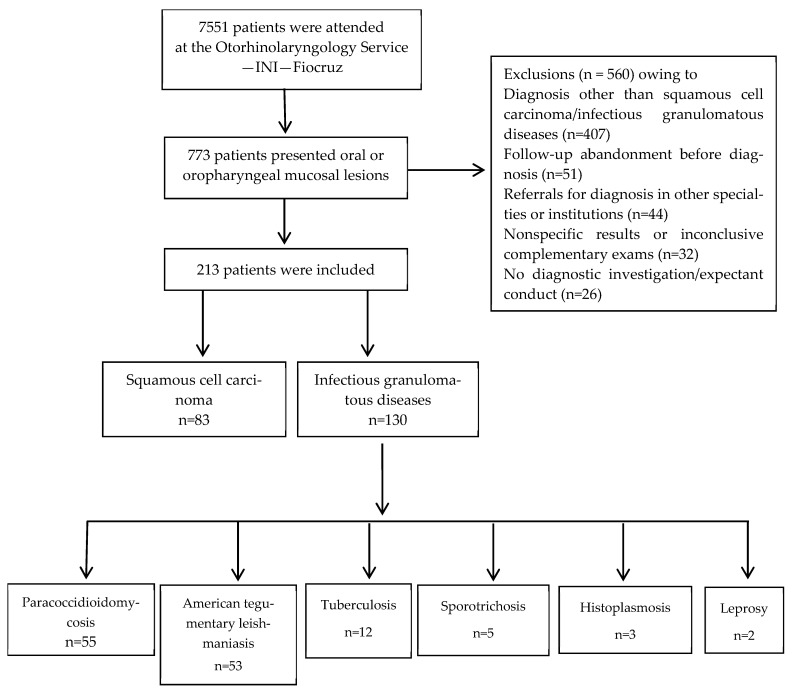
Flowchart of the selection of patients with oral or oropharyngeal lesions of squamous cell carcinoma or infectious granulomatous diseases attended at the Otorhinolaryngology Service of a reference center for infectious diseases from 2005 to 2017 (adapted from [45]).

A total of 7551 medical records were reviewed, and 213 (2.9%) patients were included in the study (Figure 1).

**Figure 2:**
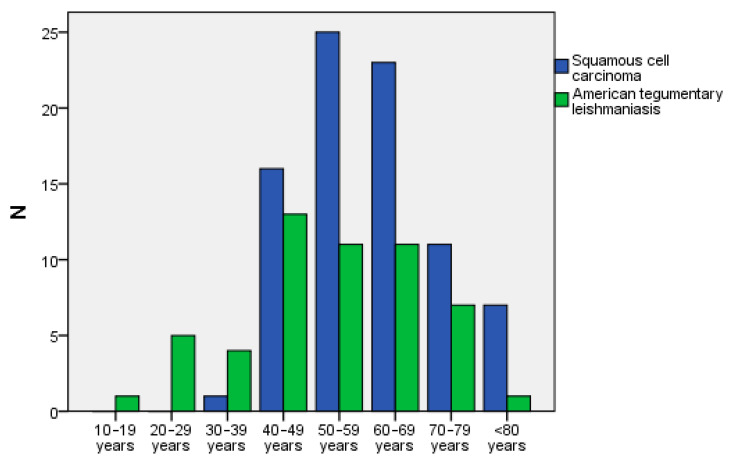
Distribution, by age group, of patients with oral and/or oropharyngeal mucosal lesions of squamous cell carcinoma and American tegumentary leishmaniasis attended at the Otorhinolaryngology Service of a reference center for infectious diseases from 2005 to 2017.

Diagnostic confirmation was obtained through histopathological examination of biopsy specimens for 175 (82.2%) patients. Diagnosis of the other patients was confirmed by serological laboratory tests, direct examination or isolation of the etiologic agent in culture, or total remission of the manifestations after specific treatment. The sociodemographic aspects of the patients are described in Figure 2, Table 1 and Table 2, and Supplemental Figure S1. The age of patients with ATL OOPML (range = 15 to 80 years, mean = 53.4 ± 16.1) and IGD OOPML (range = 15 to 80 years, mean = 50.7 ± 13.2) was lower than that of patients with SCC OOPML (range = 34 to 93 years, mean = 59.4 ± 12.4; (*p* = 0.001 and *p* < 0.001, respectively).

The results of the comparative analysis of sociodemographic variables between the IGD OOPML and SCC OOPML groups showed findings similar to those observed between the ATL OOPML and SCC OOPML groups, except for smoking, for which a significant association with SCC was observed (Supplemental Table S1).

The duration of evolution for patients with SCC OOPML (n = 74; range = 0.75 to 60 months, median = 3; IQR = 2–6) was shorter than that of those with ATL OOPML (n = 40; range = 0.75 to 60 months, median = 7; IQR = 3–22.5; *p* = 0.003) and IGD OOPML (n = 100; range = 0.25 to 120 months, median = 6; IQR = 3–12; *p* = 0.007).

**Figure 3:**
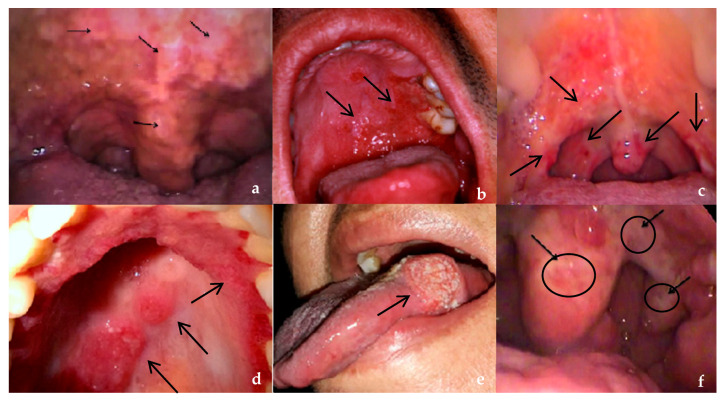
(**a**) Leprosy—granular lesions on soft palate (arrows); (**b**) paracoccidioidomycosis—mulberry-like lesions on hard palate (arrows); (**c**) tuberculosis—granular lesions on hard and soft palates and tonsillar pillars (arrows); (**d**) American tegumentary leishmaniasis—infiltrative lesions on hard palate (arrows); (**e**) squamous cell carcinoma—ulcerated lesion on tongue (arrow); (**f**) histoplasmosis—ulcerated lesions on soft palate and tonsillar pillar (arrows/circles).

As presented in Table 2, there was a significant association between ulcerated and exophytic lesions, oral-only and oropharyngeal-only general locations, “tongue” subsites, one affected oral/oropharyngeal subsite, and pain complaints in patients with SCC OOPML. On the other hand, infiltrative and granular lesions, oral/oropharyngeal general location, and lip, gum, hard and soft palate, palatine tonsil, and posterior pharyngeal wall subsites showed a significant association with ATL OOPML. Similar results were found for IGD OOPML vs. SCC OOPML, except for oral-only general location, which showed significant association with IGD OOPML and “base of tongue” subsites, which showed significant association with SCC OOPML (Supplemental Table S2). The type of lesion and location according to oral/oropharyngeal subsites for the other IGD groups are described in Table 3. All mulberry-like lesions were observed in PCM patients. Images of some OOPML are presented in Figure 3.

The effect results regarding pain complaint, general location, and number of affected oral/oropharyngeal subsites obtained by logistic regression models for SCC OOPML vs. IGD OOPML (including ATL OOPML) are described in Table 4.

The effect of the number of affected oral/oropharyngeal subsites is location-dependent and cannot be evaluated in isolation. The chances of SCC in patients presenting OOPML in only one subsite were 225% and 338% higher than for those with OOPML in >1 subsite when analyzing patients with lesions restricted to the oral cavity and oropharynx, respectively, compared to those with OOPML in both locations. Additionally, patients with pain had 3.96 times higher odds of SCC OOPML (95% CI 1.97–12.51) than those with IGD OOPML.

Patients with ATL OOPML (n = 38) and IGD OOPML (n = 66) had simultaneous nasal and/or laryngeal involvement more frequently than those with SCC OOPML (n = 3) (*p* < 0.001).

## 4. Discussion

This study was conducted using data collected from a reference center for infectious diseases and, contrary to expectation, the frequency of SCC OOPML was high. This highlights the issue regarding the difficulty of differential diagnosis of OOPML [45] and the similarities in the clinical aspects of IGDs and SCC OOPML. Although IGDs have great relevance in endemic regions, globalization has spread these diseases to previously unexposed areas. With the increase in life expectancy, older individuals are more susceptible to ageing-related diseases such as cancer; however, OOPML of infectious diseases should not be disregarded in older populations, as many of these diseases are more common in people aged over 50 years [13,41,47].

To our knowledge, there are no studies comparing ATL, other IGDs, and SCC OOPML. IGD OOPML are often misdiagnosed as SCC, not only because of the rarity of OOPML in some of these diseases or the lack of awareness of these manifestations by health professionals, but mainly because their clinical aspects are often nonspecific and varied [3,4,43,48]. Even characteristic lesions of some diseases are not always present. As an example, in 20 (36.6%) cases of PCM in the present study, the mulberry-like description considered typical of this disease was not observed, and, thus, this can hinder diagnosis, especially in regions also endemic for ATL and SP [2,13,47]. The comparative analysis between ATL OOPML and SCC OOPML was similar to the findings observed in the comparison between all IGD OOPML evaluated and SCC OOPML, highlighting the diagnostic challenge resulting from the high similarity among these infectious conditions.

SCC, ATL, and other IGD OOPML (PCM, SP, HP, TB, LP) were more often observed in males, as previously described [3,13,29,43,49]. Although the burden of SCC in women is still considered low, the number of cases in females has increased in Europe, possibly owing to changes in sexual habits and risk factors in this part of the population [41,49,50]. In females with non-habit-related SCC, hormones might play a role in the development and progression of oral SCC [51]. On the other hand, the lower incidence of some IGDs, such as ATL and PCM, in women may be related to hormonal protection [52,53].

We observed that patients with SCC OOPML had a higher mean age than those with ATL and IGD OOPML, corroborating the findings of other studies [38,47,49,54]. However, an increase in the prevalence of SCC has been observed among younger individuals in some regions, possibly because HPV-related SCC generally occurs in younger people [54,55].

In the present study, patients with OOPML in all groups had a low educational level. Lower educational levels are generally observed along with low socioeconomic status, inadequate housing, malnutrition, lack of sanitation, and poor oral health, which can facilitate the occurrence of IGDs, in addition to hindering access to medical care for diagnosis/treatment [44,56,57]. For example, ATL and PCM are neglected diseases associated with economically vulnerable populations [44,56].

Although there was underreporting of HIV co-infection in our sample, we observed that most patients with SCC, ATL, PCM, and TB OOPML (for whom this information was available) were HIV-negative. On the other hand, all the HP and 75% of patients with SP (for whom this information was available) were immunosuppressed (HIV-positive; post-transplant immunosuppressive therapy). No data on HIV co-infection in SCC and PCM were found in the literature reviewed [38,43,47,49,54]. On the other hand, published studies have observed little or no HIV co-infection in patients with ATL and TB OOPML [3,39,48,58], although the correlation between HIV-ATL and HIV-TB is well established [8,31]. The involvement of the oral/oropharyngeal mucosa and other sites of the upper aerodigestive tract in patients with HP and SP seems to be part of the disseminated disease, whereas isolated oral lesions seem to be the primary sign in immunocompetent patients [30,59,60]. It is suggested that immunosuppression facilitates the hematogenous spread of these fungi [2]. It is noteworthy that the only immunocompetent patient with SP presented a labial ulcer, which appeared after a cat scratch, indicating the probable local inoculation of the fungus.

Despite the well-established influence of smoking and alcohol use on both SCC and IGDs [41,46,61], the underreporting of information about these habits in medical records may have impaired data analysis. Even so, we found that more than half of the patients with SCC OOPML (for whom this information was available) were smokers and observed a significant association between this habit and SCC OOPML when compared to ATL patients. Although we did not observe an association between smoking and ATL, this habit has been associated with more severe forms of laryngeal tuberculosis [46]. Chewing tobacco, betel quid and areca nut, HPV infection, and poor oral health have also been associated with oral/oropharyngeal cancer [41,62,63]. Tobacco remains an important risk factor for SCC, but a decrease in smoking-associated cancers and an increase in HPV-positive cancers have been observed, particularly in young patients and in high-income countries [41,62,64]. Data on oral health could not be evaluated in our study, highlighting the importance of multidisciplinary teams for patient care.

The median duration of disease evolution was significantly shorter for patients with SCC OOPML, probably because these lesions were associated with pain, making patients seek care earlier. Conversely, the longer evolution time observed in patients with ATL and IGD OOPML may be related to the lower symptomatology, as well as the difficulty in accessing specialized care for diagnosing these diseases [8,38,44]. In addition to socioeconomic factors, the variation in the median duration of evolution of SSC is also possibly influenced by fear of diagnosis [57,65,66]. However, early diagnosis is important in reducing sequelae in both SCC and IGD [13,41].

Although we observed varied lesions in both the ATL, IGD, and SCC groups (with the exception of mulberry-like ones, which were observed exclusively in PCM), we found a significant association of ulcerated and exophytic lesions with SCC, as reported by [49,66], and of infiltrative and granular OOPML with ATL and the other IGDs. Similar to other studies, we identified a higher frequency of mulberry-like lesions in PCM [22,38,43] and ulcerated and granular lesions in TB [3,39]. However, infiltrative lesions were the most common in ATL in our sample, unlike exophytic or ulcerated ones reported by other authors [7,48,67]. We did not observe a predominant lesion type for HP, SP, and LP, possibly owing to the low frequency of OOPML in these IGDs and the small sample sizes in the present study; however, some studies have reported higher frequencies of ulcerated lesions in HP and SP [2,4]. Regarding LP, some suggest that specific LP OOPML are a rare event and manifest predominantly as plaques and infiltrative lesions, especially in multibacillary patients [6,36], while other studies report 20–90% occurrence of OOPML in this disease [37,68,69]. However, the manifestations are described as nonspecific, such as a fissured tongue and candidiasis [37,68,69]. PCM and ATL were the most frequent IGD OOPML in our sample. PCM frequently presents with oral lesions [22] and the oral cavity/oropharynx are the second most affected anatomical sites in the head and neck in ATL patients [13]. Despite OOPML being less common in ATL [48,70], we observed a similar number of them as for PCM OOPML. This is likely due to the fact that the Laboratory of Clinical Research and Surveillance in Leishmaniasis (LapClinVigiLeish), INI-Fiocruz, attends approximately 50% of ATL cases in the Rio de Janeiro state and 90% of the cases in the Rio de Janeiro metropolitan area, and also to the referral of these patients to systematic otorhinolaryngological examination in our service [71]. Importantly, despite some information available in the literature [72], no evidence of concomitant infection with *Leishmania* spp. or any of the other IGDs evaluated in association with tumor cells was observed in this series.

In our study, the effects of the number of oral/oropharyngeal subsites and pain complaints remained even after adjusting for other variables. The effect of the number of oral/oropharyngeal sites in the outcomes (IGD vs. SCC) exists but is dependent on the general location of OOPML (oral only, oropharyngeal only, or oral/oropharyngeal). Patients with OOPML in only one oral or oropharyngeal subsite were more likely to have SCC than IGDs when compared to those with lesions in both locations. Additionally, patients presenting pain complaints were significantly more likely to have SCC OOPML. This symptom was associated with SCC, probably due to the fact that ulcers were the most frequent OOPML in this disease, while most ATL and other IGD cases were associated with infiltrative and granular lesions. As mentioned, although we could not find comparative analyses of SCC and IGD OOPML in the literature, some specific studies on SCC and IGDs report the occurrence of pain, while asymptomatic patients were more frequent in others [2,39,43,47,73,74]. Nevertheless, unlike ours, most of these studies used descriptive models rather than multiple covariate analysis. Differences in symptomatology might be attributed to, in addition to the type of lesion, the location of OOPML and early treatment of initial lesions.

Lesions on the tongue or base of tongue were associated with SCC, while lesions in the subsites of the lips, gum, hard and soft palates, tonsil, and posterior pharyngeal wall, with simultaneous involvement of other sites of the upper aerodigestive tract, showed a significant association with ATL and other IGDs. Similar to other reports, the tongue was the most frequent subsite of OOPML in SCC, although in contrast to those reports, the floor of mouth, buccal mucosa, and gum were little affected by SCC OOPML in our sample [49,54,57]. The variation between the most frequent subsites can likely be attributed to habits/geographic regions (e.g., tobacco and betel quid chewing, in which the buccal mucosa has greater contact with the carcinogenic substances) [55,75].

We found that the gums were the most affected site in PCM, followed by the hard palate and oropharynx. This subsite is also the most frequent in other studies, but typically followed by the lip/labial commissure and buccal mucosa [22,38,43,47]. OOPML were more frequent in the soft and hard palate and oropharynx of patients with ATL, as already reported [47,58,67]. Unlike other studies, there were no cases of tongue lesions in TB in our sample [3,32,33]; instead, the hard palate was the only oral subsite affected in these patients. The other subsites were all oropharyngeal. We also did not observe a predominant subsite for the OOPML of HP, SP, and LP [2,4,26,36].

**Figure 4:**
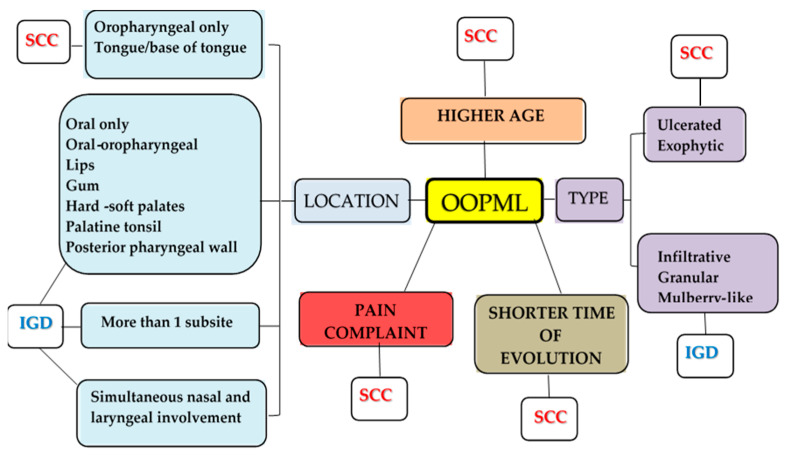
Fluxogram of diagnostic hypotheses of oral/oropharyngeal mucosal lesions of patients with oral and/or oropharyngeal mucosal lesions of infectious granulomatous diseases (including American tegumentary leishmaniasis) and squamous cell carcinoma attended at an Otorhinolaryngology Service of a reference center for infectious diseases from 2005 to 2017. OOPML—oral and/or oropharyngeal mucosal lesions; SCC—squamous cell carcinoma; IGDs—infectious granulomatous diseases.

To the best of our knowledge, this study is unique in comparing ATL, other IGDs, and SCC OOPML. Although the number of patients in our cohort does not allow the creation of a predictive model, our findings could help health professionals in the diagnostic investigation of OOPML. As summarized in Figure 4, our results show that pain complaints and the presence of OOPML in only one oral or oropharyngeal subsite are more likely to occur in cases of SCC OOPML. Ulcerated and exophytic lesions located on the tongue or base of tongue and OOPML in older individuals with shorter duration of evolution suggest SCC. On the other hand, infiltrative, granular, and mulberry-like OOPML, located only in the oral cavity or in the oral cavity and oropharynx, at the subsites of the lips, gum, hard and soft palates, tonsil, and posterior pharyngeal wall, suggest ATL and other IGDs. Moreover, the involvement of more than one oral/oropharyngeal subsite per patient increases the chances of IGD OOPML by 2.2 times. Simultaneous nasal and/or laryngeal involvement was also associated with IGD; therefore, the evaluation of other upper aerodigestive sites could aid diagnosis. Despite the difficulty in making a diagnosis based on clinical signs and symptoms of OOPML, our study highlights some information that may assist health professionals in considering and investigating these diseases, thus facilitating an early diagnosis and faster implementation of therapy with consequent reduction of sequelae.

## Figures and Tables

**Table 1:** Epidemiological characteristics of patients with oral and/or oropharyngeal mucosal lesions of squamous cell carcinoma and American tegumentary leishmaniasis attended at the Otorhinolaryngology Service of a reference center for infectious diseases from 2005 to 2017.

		Squamous Cell Carcinoma (SCC)		American Tegumentary Leishmaniasis (ATL)		*p*-Value *
		n	%	n	%	
Sex (n = 136)	Female	17	20.5	7	13.2	0.278
	Male	66	79.5	46	86.8	
Race (n = 100)	White	25	53.2	31	29.7	0.594
	Not white	22	46.8	22	41.5	
Education level (n = 118)	Until junior high	49	73.1	41	80.4	0.359
	High school and above	18	26.9	10	19.6	
HIV co-infection (n = 56)	Negative	26	92.9	21	75.0	0.069
	Positive	2	7.1	7	25.0	
Smoking (n= 99)	No	26	34.7	18	75.0	0.001
	Yes	49	65.3	6	25.0	
Alcohol use (n = 47)	No	20	60.6	10	71.4	0.480
	Yes	13	39.4	4	28.6	

* Pearson’s chi-square test.

**Table 2:** Clinical characteristics of patients with oral and/or oropharyngeal mucosal lesions of squamous cell carcinoma and American tegumentary leishmaniasis attended at the Otorhinolaryngology Service of a reference center for infectious diseases from 2005 to 2017.

		Squamous Cell Carcinoma (SCC)		American Tegumentary Leishmaniasis (ATL)		*p*-Value *
		n	%	n	%	
Type of lesion ^1,2^ n = 127	Ulcerated	53	70.7	14	26.9	<0.001
	Exophytic	24	32	7	13.5	0.017
	Infiltrative	18	24	35	67.3	<0.001
	Granular	12	16	24	46.2	<0.001
	Hyperemic	2	2.7	6	11.5	0.063
	1 type of lesion	42	56	23	44.2	0.192
	>1 type of lesion	33	44	29	55.8	
General location n = 136	Oral only	34	41.0	19	35.8	0.023
	Oropharyngeal only	37	44.6	16	30.2	
	Oral/oropharyngeal	12	14.5	18	34.0	
Number of affected oral/oropharyngeal subsites n = 134	1 subsite	49	60.5	21	39.6	0.018
	>1 subsite	32	39.5	32	60.4	
Subsites ^3^ n = 134	Lips	5	6.2	14	26.4	0.001
	Gum	5	6.2	12	22.6	0.005
	Buccal mucosa	7	8.6	1	1.9	0.146
	Tongue	22	27.2	1	1.9	<0.001
	Floor of mouth	2	2.5	0	0	**
	Hard palate	10	12.3	23	43.4	<0.001
	Base of tongue	7	8.6	0	0	**
	Soft palate	23	28.4	30	56.6	0.001
	Tonsillar pillars ^4^	20	24.7	11	20.8	0.597
	Palatine tonsils ^5^	26	32.1	6	11.3	0.006
	Posterior pharyngeal wall	4	4.9	15	28.3	<0.001
Pain complaint n = 97	Yes	56	83.6	11	36.7	<0.001
	No	11	16.4	19	63.3	

^1^ Possibly more than one type of lesion per patient; ^2^ the detailed description of the lesion types is described in the methods section; ^3^ possibly more than one subsite per patient; ^4^ anterior and posterior tonsillar pillars; ^5^ tonsil or tonsillar pocket; * Pearson’s chi-square test for all variables, except for “hyperemic”, “buccal mucosa”, and “tongue” (Fisher’s exact test); ** statistical test could not be performed as cell count = 0.

**Table 3:** Type of lesion and location according to oral/oropharyngeal subsites of patients with infectious granulomatous diseases (other than American tegumentary leishmaniasis) attended at the Otorhinolaryngology Service of a reference center for infectious diseases from 2005 to 2017.

		PCM ^1^ (n = 55)	TB ^2^ (n= 12)	SP ^3^ (n= 5)	HP ^4^ (n = 3)	LP ^5^ (n = 2)
	n	n = 54 (94.8%)	n = 12 (100%)	n = 4 (80%)	n = 2 (66.6%)	n = 2 (100%)
Type of lesion ^6,7^	Ulcerated	17 (31.5%)	6 (50%)	1 (25%)	*	**
	Exophytic	2 (3.7%)	–	–		
	Infiltrative	10 (18.5%)	3 (25%)	1 (25%)		
	Granular	7 (12.7%)	7 (58.3%)	2 (50%)		
	Hyperemic	8 (14.5%)	–	–		
	Mulberry-like	35 (64.8%)	–	–		
	n	n = 55 (100%)	n = 11 (91.7%)	n = 5 (100%)	n = 2 (66.6%)	n = 2 (100%)
Subsites ^8^	Lips	16 (29.1%)	–	2 (40%)		
	Gum	24 (43.6%)	–	1 (20%)		
	Buccal mucosa	11 (20%)	–	–		
	Tongue	17 (30.9%)	–	–		
	Floor of mouth	5 (9.1%)	–	–		
	Hard palate	15 (27.3%)	2 (18.2%)	3 (60%)		
	Base of tongue	2 (3.6%)	1 (9.1%)	–		
	Soft palate	18 (32.7%)	6 (54.5%)	2 (40%)		
	Tonsillar pillars	14 (25.5%)	4 (36.4%)	–		
	Palatine tonsils	9 (16.4%)	–	–		
	Posterior pharyngeal wall	4 (7.3%)	–	2 (40%)		

^1^ Paracoccidioidomycosis; ^2^ tuberculosis; ^3^ sporotrichosis; ^4^ histoplasmosis; ^5^ leprosy; ^6^ the detailed description of the lesion types is described in the methods section; ^7^ may be more than one type of lesion per patient; ^8^ may be more than one subsite per patient; * both presented ulcerated and exophytic lesions on the gum and hard palate in one patient and on the hard and soft palate in the other; ** one patient presented granular lesions in the hard and soft palate and the other had infiltrative and granular lesions in the tongue and soft palate.

**Table 4:** Crude and adjusted effects of variables according to the outcome occurrence of squamous cell carcinoma in 213 patients attended at the Otorhinolaryngology Service of a reference center for infectious diseases from 2005 to 2017.

	Unadjusted OR *	95% CI **		Adjusted OR ***	95% CI	
Oropharyngeal only location	1.89	0.99	3.60	-	-	-
Only 1 subsite per patient	2.47	1.40	4.37	-	-	-
Interaction						
Only Oral: Only 1 subsite per patient	-	-	-	3.25	1.22	8.67
Oropharyngeal only: Only 1 subsite per patient	-	-	-	4.88	1.61	14.83
Pain complaint	4.39	2.01	9.57	4.96	1.97	12.51

* odds ratio; ** confidence interval; *** adjustment of the effect of variables on the outcome based on age, sex, and duration of evolution.

## Data Availability

The original contributions presented in this study are included in the article/Supplementary Material. Further inquiries can be directed to the corresponding author.

## Supplementary Materials

The following supporting information can be downloaded at https://www.mdpi.com/article/10.3390/pathogens15010101/s1: Figure S1: Distribution, by age group, of patients with oral and/or oropharyngeal mucosal lesions of squamous cell carcinoma and infectious granulomatous diseases attended at the Otorhinolaryngology Service of a reference center for infectious diseases from 2005 to 2017; Table S1: Epidemiological characteristics of patients with oral and/or oropharyngeal mucosal lesions of squamous cell carcinoma and infectious granulomatous diseases attended at the Otorhinolaryngology Service of a reference center for infectious diseases from 2005 to 2017; Table S2: Clinical characteristics of patients with oral and/or oropharyngeal mucosal lesions of squamous cell carcinoma and infectious granulomatous diseases attended at the Otorhinolaryngology Service of a reference center for infectious diseases from 2005 to 2017.

## Abbreviations

The following abbreviations are used in this manuscript:

ATL	American tegumentary leishmaniasis
IGD	infectious granulomatous disease
OOPML	oral/oropharyngeal mucosal lesions
SCC	squamous cell carcinoma
PCM	paracoccidioidomycosis
SP	sporotrichosis
HP	histoplasmosis
TB	tuberculosis
LP	leprosy
PAHO	Pan American Health Organization
HPV	human papillomavirus
UV	ultraviolet
SD	standard deviation
IQR	interquartile range
OR	odds ratio
CI	confidence intervals
