# Supplementary material for: Comparative Analysis of Oral and Oropharyngeal Mucosal Lesions of American Tegumentary Leishmaniasis and Other Infectious Granulomatous Diseases and Squamous Cell Carcinoma
Source: Pathogens. 2026 Jan 17;15(1):101. doi: 10.3390/pathogens15010101 (PMC12844808; doi:10.3390/pathogens15010101)
Supplement: Supplementary file 1 [file pathogens-15-00101-s001.zip › pathogens-4089872-supplementary.pdf]

pathogens

MDPI

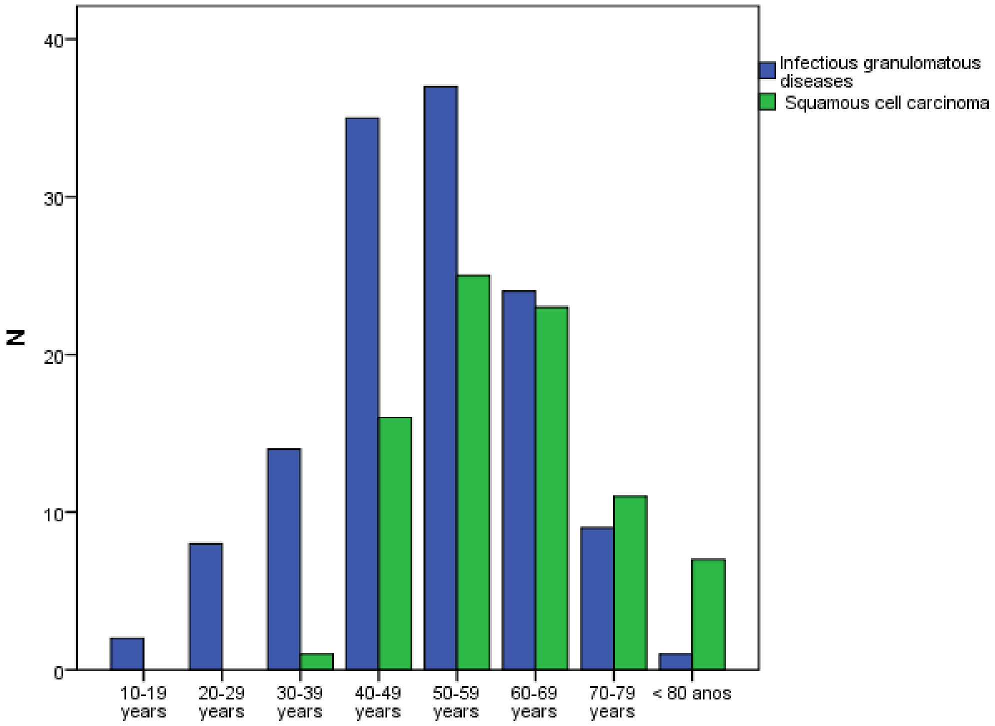

Supplemental Figure S1 - Distribution, by age group, of patients with oral and/or oropharyngeal mucosal lesions of squamous cell carcinoma and infectious granulomatous diseases attended at the Otorhinolaryngology Service of a reference center for infectious diseases from 2005 to 2017.

Supplemental Table S1 - Epidemiological characteristics of patients with oral and/or oropharyngeal mucosal lesions of squamous cell carcinoma and infectious granulomatous diseases attended at the Otorhinolaryngology Service of a reference center for infectious diseases from 2005 to 2017.

		Squamous cell carcinoma		Infectious granulomatous diseases		p value*
		n	%	n	%	
**Sex (N=213)**	Female	17	20.5	21	16.2	0.465
	Male	66	79.5	109	88.3	
**Skin color (N=175)**	White	25	53.2	60	46.9	0.498
	Not white	22	46.8	68	53.1	
**Education level (N=193)**	Until junior high	49	73.1	106	84.1	0.087
	High school and above	18	26.9	20	15.9	
**HIV co-infection (N=98)**	Negative	26	92.9	54	77.1	0.084
	Positive	2	7.1	16	22.9	
**Smoking (N=159)**	No	26	34.7	34	40.5	0.513
	Yes	49	65.3	50	59.5	
**Alcohol use (N=94)**	No	20	60.6	38	62.3	1.000
	Yes	13	39.4	23	37.7	

Supplemental Table S2 - Clinical characteristics of patients with oral and/or oropharyngeal mucosal lesions of squamous cell carcinoma and infectious granulomatous diseases attended at the Otorhinolaryngology Service of a reference center for infectious diseases from 2005 to 2017.

		**Squamous cell carcinoma (SCC)**		**Infectious granulomatous diseases (IGD)**		**p value***
		**N**	**%**	**n**	**%**	
**Type of lesion[1] N=201**	Ulcerated	53	70.7	40	31.7	**<0.001**
	Exophytic	24	32	11	8.7	**<0.001**
	Infiltrative	18	24	50	39.7	**0.031**
	Granular	12	16	42	33.3	**0.008**
	Hyperemic	2	2.4	14	11.1	0.056
	Mulberry-like	0	0	35	27.8	**
	1 type of lesion	42	56	74	58.7	0.768
	>1 type of lesion	33	44	52	41.3	
**General location N=213**	Oral only	34	41.0	54	41.5	**0.001**
	Oropharyngeal only	37	44.6	31	26.8	
	Oral/oropharyngeal	12	14.5	45	34.8	
**Number of affected oral/oropharyngeal subsites N=209**	1 subsite	49	60.5	49	38.3	**0.002**
	>1 subsite	32	39.5	79	61.7	
**Subsites[2] N=209**	Lips	5	6.2	32	25	**<0.001**
	Gum	5	6.2	38	29.7	**<0.001**
	Buccal mucosa	7	8.6	12	9.4	0.857
	Tongue	22	27.2	19	14.8	**0.033**
	Floor of mouth	2	2.5	5	3.9	0.709
	Hard palate	10	12.3	46	35.9	**<0.001**
	Base of tongue	7	8.6	3	2.3	**0.049**
	Soft palate	23	28.4	59	46.1	**0.013**
	Tonsillar pillars[3]	20	24.1	29	22.7	0.740
	Palatine tonsils[4]	26	32.1	22	17.2	**0.018**
	Posterior pharyngeal wall	4	4.9	25	19.5	**0.003**
**Pain complaint N=149**	Yes	56	83.6	44	53.7	**<0.001**
	No	11	16.4	38	46.3	

[1]May be more than one type of lesion per patient; [2]may be more than one subsite per patient; [4]anterior and posterior tonsillar pillars; [5]tonsil or tonsillar pocket; *Pearson's chi-square test for all variables, except for "floor of mouth" and "base of tongue" (Fisher's exact test); **statistical test could not be performed as cell count=0.
